# Y Chromosome Lineage- and Village-Specific Genes on Chromosomes 1p22 and 6q27 Control Visceral Leishmaniasis in Sudan

**DOI:** 10.1371/journal.pgen.0030071

**Published:** 2007-05-11

**Authors:** E. Nancy Miller, Manal Fadl, Hiba S Mohamed, Abier Elzein, Sarra E Jamieson, Heather J Cordell, Christopher S Peacock, Michaela Fakiola, Madhuri Raju, Eltahir A Khalil, Ahmed Elhassan, Ahmed M Musa, Muntaser E Ibrahim, Jenefer M Blackwell

**Affiliations:** 1 Cambridge Institute for Medical Research, University of Cambridge School of Clinical Medicine, Cambridge, United Kingdom; 2 Institute of Endemic Diseases, University of Khartoum, Khartoum, Sudan; Stanford University, United States of America

## Abstract

Familial clustering and ethnic differences suggest that visceral leishmaniasis caused by Leishmania donovani is under genetic control. A recent genome scan provided evidence for a major susceptibility gene on Chromosome 22q12 in the Aringa ethnic group in Sudan. We now report a genome-wide scan using 69 families with 173 affected relatives from two villages occupied by the related Masalit ethnic group. A primary ten-centimorgan scan followed by refined mapping provided evidence for major loci at 1p22 (LOD score 5.65; nominal *p* = 1.72 × 10^−7^; empirical *p* < 1 × 10^−5^; λ_S_ = 5.1) and 6q27 (LOD score 3.74; nominal *p* = 1.68 × 10^−5^; empirical *p* < 1 × 10^−4^; λ_S_ = 2.3) that were Y chromosome–lineage and village-specific. Neither village supported a visceral leishmaniasis susceptibility gene on 22q12. The results suggest strong lineage-specific genes due to founder effect and consanguinity in these recently immigrant populations. These chance events in ethnically uniform African populations provide a powerful resource in the search for genes and mechanisms that regulate this complex disease.

## Author Summary

The parasitic disease kala-azar, or visceral leishmaniasis, is associated with liver, spleen, and lymph gland enlargement, as well as fever, weight loss, and anaemia. It is fatal unless treated. Three major foci of disease occur in India, Sudan, and Brazil. Importantly, 80%–90% of infections are asymptomatic. Understanding why two people with the same exposure to infection differ in susceptibility could provide important leads for improved therapies. We studied families with multiple cases of clinical disease from two villages in Sudan. After typing 300–400 genetic markers across the human genome, we determined which chromosomes carry susceptibility genes. We were surprised that our results differed from those published earlier for a village 100 kilometers from our site. All of these villages are occupied by people of the same ethnic group who migrated from western Sudan late last century following a major drought. We stratified our analysis by village, and used male Y chromosome markers to tag extended pedigrees. Our results suggest that recent immigration, in combination with consanguineal marriage in a strongly patriarchal society, has amplified founder effects resulting in different lineages within each village carrying different susceptibility loci. This demonstrates the importance of understanding population genetic substructure in studying genes that regulate complex disease.

## Introduction

Ninety percent of clinical visceral leishmaniasis (VL) cases caused by protozoa of the L. donovani species complex (L. donovani, L. archibaldi, *L. infantum,* and L. chagasi) occur in three foci in India/Bangladesh/Nepal, Sudan, and Brazil. Skin-test data and lymphocyte proliferation assays [1–4] indicate that only one in 5–10 infected individuals develop clinical disease. Familial aggregation is a feature of VL in Brazil [5], providing a high relative risk (λ_2S_ = 34) of disease in further siblings of affected sibling pairs [6]. In Sudan, familial clustering and marked differences in incidence of clinical disease and skin-test reactivity between villages inhabited by different ethnic groups that share environment and exposure [7,8] support a contribution of host genotype to susceptibility. In mice [9], different genes control innate versus adaptive immune responses, and we expect genetic control in humans to be complex. Nevertheless, understanding the genes/mechanisms that determine why two people with the same exposure differ in susceptibility to VL could provide important leads for improved therapies. A number of candidate gene studies have been undertaken that support roles for polymorphisms at *SLC11A1*, *IL4,* and *IFNGR1* in controlling susceptibility to visceral leishmaniasis or post kala-azar dermal leishmaniasis in Sudan [10–12]. A genome-wide scan recently undertaken in eastern Sudan by Bucheton et al. [13] also reported a major gene (LOD score 3.5; *p* = 3 × 10^−5^) on Chromosome 22q12 controlling VL in the Aringa ethnic group. We now report on a second genome-wide scan undertaken in two villages occupied by the related Masalit ethnic group in eastern Sudan, in which we provide evidence for major loci at 1p22 and 6q27 that are Y chromosome–lineage and village-specific. Neither village provided evidence for a VL susceptibility gene on 22q12. The results suggest strong lineage-specific genes due to founder effect and consanguinity in these recently immigrant populations, and point to the potential power these chance events in ethnically uniform African populations provide in the search for genes and mechanisms that regulate this complex disease.

## Results

### Primary Genome Scan

**Figure 1:**
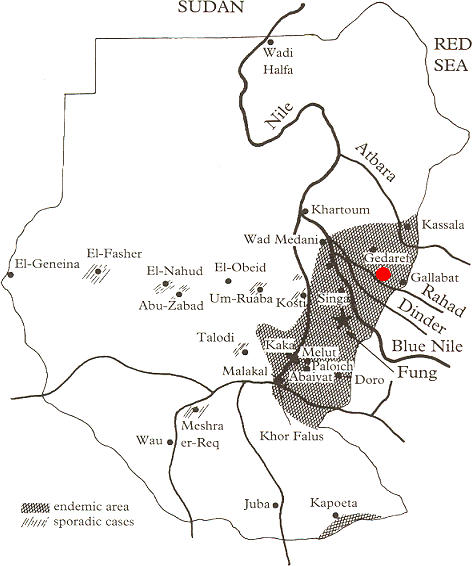
Map of Sudan Showing Locations of Viseral Leishmaniasis Study Sites in Sudan. Locations of El-Rugab and Um-Salala villages (red dot) used in this study are shown in relation to Barbar El Fugura village (near Gedaref) used in the earlier study of Bucheton et al. [13]. Areas endemic for visceral leishmaniasis, or where sporadic cases occur, are marked on the map. Modified with permission from Zijlstra et al. [34].

**Figure 2:**
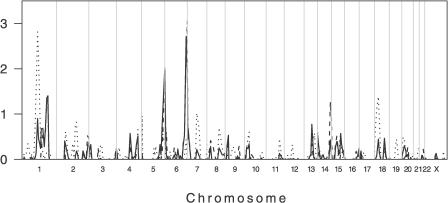
Primary Genome-wide Linkage Scan of 219 Individuals Belonging to 38 Extended Multicase Pedigrees from Sudan Dotted and dashed lines show the data stratified by Um-Salala and El-Rugab, respectively. The solid line represents the combined analysis across both villages. Multipoint LOD scores (plotted as sign(dhat)•LOD [36]) are shown on the vertical axis and the distance in cM (Genethon Map) from the p terminus for all chromosomes on the horizontal axis. As a guide to evaluating significance of genome-wide data, Lander and Kruglyak [14] propose threshold point-wise LOD scores of 2.2 (nominal *p* value 7.4 × 10^−4^) for “suggestive linkage” and 3.6 (*p* = 2.2 × 10^−5^) for “significant linkage.” Refined mapping reduced evidence for linkage on Chromosome 5 and eliminated evidence for linkage on Chromosome 13 (data not shown). In addition to the major village-specific peaks of linkage on Chromosomes 1 and 6 (cf. main text), evidence for novel village-specific regions of linkage (at *p* < 0.01) not seen in the unstratified analysis of the original primary genome scan data was also obtained at Chromosomes 14q32.2 (D14S65; LOD score 1.26; *p* = 0.008) for El-Rugab, and 18p11.22 (D18S464 ; LOD score 1.38; *p* = 0.005) for Um-Salala. Refined mapping reduced evidence for linkage on Chromosome 14 and eliminated evidence for linkage on Chromosome 18 (data not shown). Evidence for village-specific linkage was also observed at D2S142 (LOD score 0.82; *p* = 0.026) for Um-Salala. We added more markers in this region because it coincided precisely with a second peak of linkage (LOD score 2.29; *p* = 5.86 × 10^−4^) reported by Bucheton et al. [13] for Aringa families negative for linkage at D22S280 on 22q12, providing further support for village- and possibly lineage-specific effects (cf. main text). In Um-Salala, the evidence for linkage to 2q23-q24 was retained (LOD score 0.92; *p* = 0.02) on refined mapping, but the peak of linkage moved 7 cM proximal to marker D2S2275. After we had demonstrated the influence of stratifying analysis by Y chromosome haplotype on village-specific peaks at 1p22 (cf. Figure 3) and 6q27 (cf. Figure 4), we went back and analysed the primary genome scan data across both villages stratifying by Y chromosome haplotype. No novel peaks for either E3b1 or A3b2 families analysed across both villages were observed (data not shown).

In their study, Bucheton et al. [13] used 63 multicase families from members of the Aringa ethnic group living in Barbar El Fugura, Gedaref State, eastern Sudan (Figure 1). The Aringa people in this village migrated from western Sudan/Chad to settle as subsistence farmers in the 1940s [13]. We worked [11,12] in two villages, El-Rugab and Um-Salala, located ~40 km apart in Galabat Province, Gadaref State, ~100 km south of Barbar El Fugura [13]. El-Rugab and Um-Salala are occupied by Nilosaharan speaking Masalit, who also migrated from western Sudan, starting in 1969, to occupy villages in the heart of the endemic area in eastern Sudan. The two villages have high rates of clinical VL. Using 38 pedigrees (48 nuclear families; Table 1), we performed a 360 microsatellite ~10-cM genome-wide scan. Lander and Kruglyak [14] proposed a classification for reporting the results of genome-wide scan data based on the number of times one would expect to see a result at random in a dense, complete genome scan. The thresholds they propose are: “suggestive linkage,” where statistical evidence would be expected to occur one time at random in a genome scan; “significant linkage,” 0.05 times; “highly significant linkage,” 0.001 times; and “confirmed linkage,” where significant linkage from an initial scan has been confirmed with a nominal *p* value of ≤0.01 in a second independent study. In the case of a sib-pair study, the first three categories correspond to point-wise significance levels of 7 × 10^−4^, 2 × 10^−5^, and 3 × 10^−7^ (LOD scores 2.2, 3.6, and 5.4). To analyze our genome-wide scan data we used nonparametric linkage analysis comparing identity-by-descent allele sharing across all relative pairs in the extended pedigrees. The thresholds proposed by Lander and Kruglyak vary marginally for other relative pairs combinations [14]. Although other authors consider these thresholds to be overly conservative [15], they serve as a guide to evaluate the significance associated with the nominal point-wise *p* values provided here. All *p* values reported here are nominal one-sided *p* values, except where we state that we have carried out simulations to calculate empirical *p* values. The nonparametric linkage analysis for the primary genome scan in Sudan provided evidence for linkage of VL to four regions on Chromosomes 1, 5, 6, and 13 (LOD scores 0.77 to 2.83; *p* values 1.5 × 10^−4^ < *p* < 0.029) across El-Rugab and Um-Salala (Figure 2). None of these matched regions that were identified in the earlier [13] primary scan of 38 families. As observed for the Masalit in our area [7,8], the Aringa ethnic group is highly susceptible to VL compared to the neighboring Hawsa and Fellata ethnic groups [13], and is closely related to the Masalit. Our failure to observe overlap with the earlier [13] study led us to consider whether founder effect and population substructure could influence genes regulating VL in different villages in eastern Sudan.

**Table 1:**
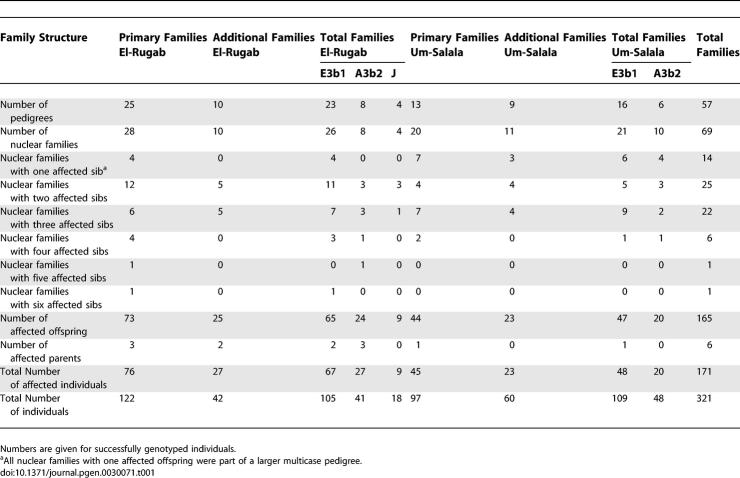
Family Structure and Distribution of Clinical VL by Village, and by Y Haplogroup within Villages, for Families Used in the Primary Genome Scan and Refined Mapping in Sudan

**Figure 3:**
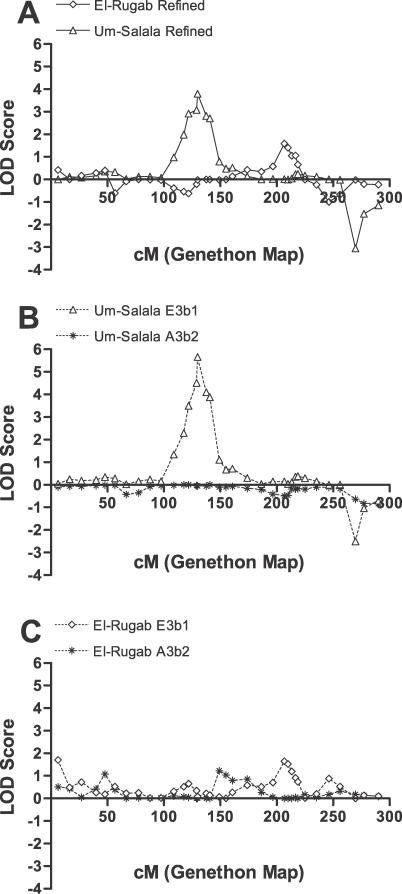
Genome Scan Data for Chromosome 1 for Multicase Families of Visceral Leishmaniasis from Sudan (A) refined mapping data by village; (B) refined map stratified by Y chromosome haplotype for Um-Salala; (C) refined map stratified by Y chromosome haplotype for El-Rugab. Multipoint LOD scores (plotted as sign(dhat)•LOD[36]) are shown on the vertical axis and the distance in cM (Genethon Map) from the p terminus on the horizontal axis. To ensure that allele-sharing LOD scores were not inflated or biased by particular individuals or families, we recalculated LOD scores randomly, dropping one pedigree at each analysis. The mean ± SD peak LOD score at 1p22 was 5.17 ± 0.83 (range 3.97–7.08), indicating most E3b1 pedigrees contribute to this linkage peak. Information contents over the region of linkage at 1p22 (114–135 cM) were (mean ± SD) 0.71±0.03 (range 0.72–0.82) for Um-Salala refined map in (A), 0.74 ± 0.05 (range 0.69–0.84) for Um-Salala E3b1 families in (B), and 0.59 ± 0.04 (range 0.56–0.65) for El-Rugab E3b1 families. Information contents over the region of linkage at 1q31 (207–219 cM) were 0.56 ± 0.05 (range 0.50–0.63) for El-Rugab refined map in (A), and 0.61 ± 0.04 (range 0.56–0.66) for Um-Salala E3b1 families in (B), and 0.56 ± 0.07 (range 0.47–0.62) for El-Rugab E3b1 families in (C).

**Figure 4:**
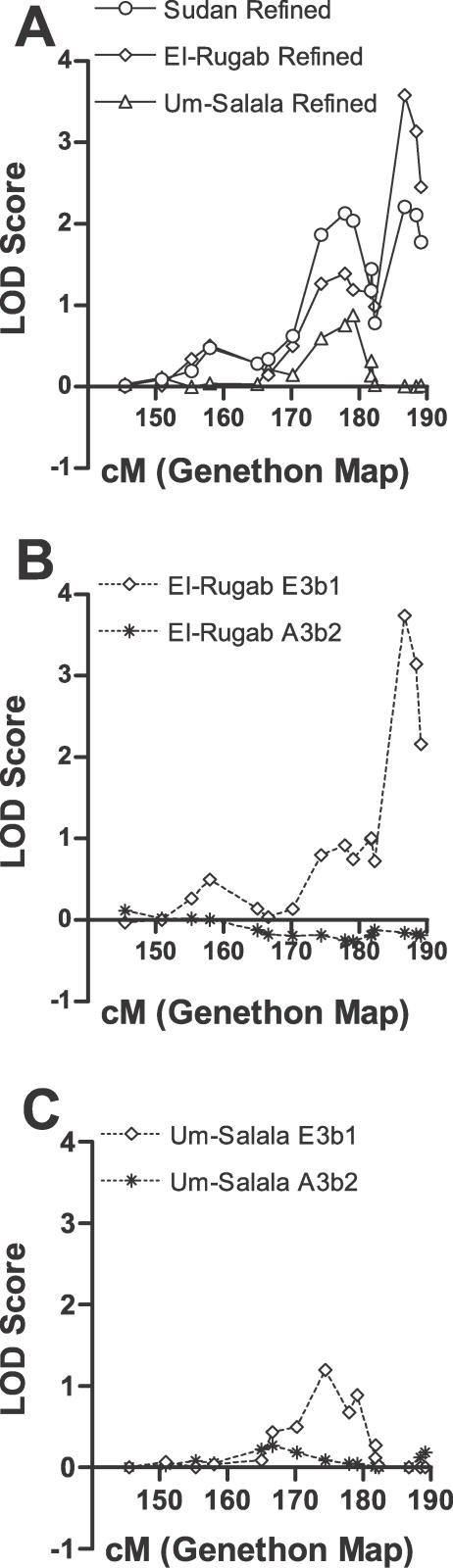
Genome Scan Data for Chromosome 6 for Multicase Families of Visceral Leishmaniasis from Sudan (A) refined mapping data by village; (B) data for El-Rugab village stratified by Y chromosome haplotype; (C) data for Um-Salala village stratified by Y chromosome haplotype. Multipoint LOD scores (plotted as sign(dhat)•LOD [36]) are shown on the vertical axis and the distance in cM (Genethon Map) from the p terminus on the horizontal axis. The mean ± SD peak LOD score at 6q27 obtained when randomly dropping one pedigree at each analysis (see legend to Figure 2) was 3.61 ± 0.46 (range 2.39–4.28). Information contents over the region of linkage at 6q27 (174–180 cM) were 0.66 ± 0.05 (range 0.61–0.72) for El-Rugab refined map in (A), 0.64 ± 0.07 (range 0.56–0.73) for El-Rugab E3b1 families in (B), and 0.87 ± 0.04 (range 0.83–0.94) for Um-Salala E3b1 families in (C).

### Estimating Consanguinity, Refined Mapping, and Stratification by Village

The Masalit ethnic group is polygamous; males have up to four wives at any time, depending on socio-economic status. The founding population for each village comprised 10–15 related males with their families. By 1997 population sizes were ~2,000 in El-Rugab and ~1,200 in Um-Salala. Using the program Pedigree Relationship Statistical Test (PREST) [16] to estimate relatedness between individuals we found that ~43% of marriages in El-Rugab and ~33% within Um-Salala were consanguineous, with varying degrees of relatedness between parents (Table 2). The combination of chance events in the founder population and consanguineal marriages could affect the genetic composition of each village. When the primary genome scan data for Sudan was stratified by village, the bimodal linkage on Chromosome 1 (Figure 2) resolved into two clear village-specific peaks: at 1p22 for Um-Salala (peak LOD score at D1S2868 = 2.81; *p* = 1.62 × 10^−4^) and 1q31.3 for El-Rugab (peak LOD score at D1S238 = 1.31; *p* = 0.007). Refined mapping provided genome-wide [14] evidence (LOD score 3.8; *p* = 1.45 × 10^−5^) for a major susceptibility gene at D1S2868 on 1p22 in Um-Salala, and improved the evidence (LOD score 1.59; *p* = 0.003) for linkage at D1S238 on 1q31.3 in El-Rugab (Figure 3A). Similarly, stratification and refined mapping on 6q27 (Figure 4A) provided evidence for a common susceptibility locus at D6S1719 (LOD score 2.13; *p* = 8.74 × 10^−4^) affecting both villages, with genome-wide [14] significance (LOD score 3.58; *p* = 2.47 × 10^−5^) for a susceptibility gene at D6S281 in El-Rugab alone.

**Table 2:**
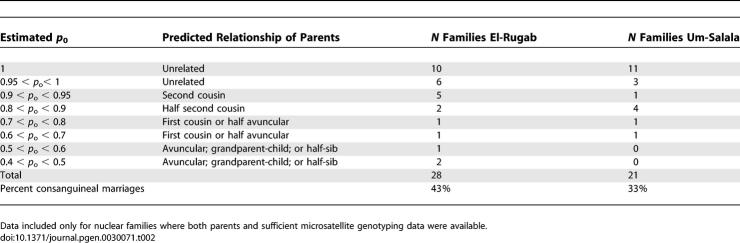
Results of PREST Analysis Undertaken to Determine the Degree of Relatedness between Parents within Families from El-Rugab and Um-Salala

To determine the contribution that loci at 1p22 and 6q27 make to genetic susceptibility to VL in each village, we estimated [17] the locus-specific λ_S_ from the ratio of the expected (0.25) proportion of affected sibpairs sharing zero alleles identical-by-descent under no linkage to the observed proportion. This ratio indicates the risk to siblings of affected individuals compared to the general population risk. At 1p22 in Um-Salala the λ_S_ was 2.90, and 1.63 at 6q27 in El-Rugab. Thus, these loci make an appreciable contribution to the total genetic component of disease susceptibility in each village, equivalent to or greater than that observed for the major genes for VL [13] at 22q12 (λ_S_ = 1.83 for all families; 2.11 for families affected towards the beginning of an outbreak), or for leprosy reported from genome scans at 10p13 in India (λ_S_ = 1.66) [18] and 6q25 in Vietnam (λ_S_ = 2.21) [19].

### Population Substructure and Analysis Allowing for Over-Relatedness in Families

To understand more about population substructure within villages, we genotyped Y chromosome markers [20] for all male family members. In all cases, all available males within a pedigree carried the same Y chromosome haplotype. Two main Y haplotypes [20,21] were present, A3b2 from haplogroup A and E3b1 from haplogroup E, shown by pedigree in Table 1. Stratifying the linkage analysis by Y chromosome haplotype demonstrated strong lineage-specific effects within each village. The peak at 1p22 in Um-Salala was contributed to only by E3b1 lineages (Figure 3B); peak LOD score was 5.65 (*p* = 1.7 × 10^−7^), with locus-specific λ_S_ 5.07 at D1S2868. Consistent with the result obtained using stratification by village, neither E3b1 nor A3b2 lineages from El-Rugab village showed linkage to disease at 1p22 (Figure 3C). The peak at 6q27 (Figure 4B) for El-Rugab was also specific to E3b1 families; peak LOD score was 3.74 (*p* = 1.68 × 10^−5^), locus-specific λ_S_ 2.31 at D6S281. Again, neither E3b1 nor A3b2 lineages from Um-Salala village showed linkage to disease at the major peak at D6S281 (Genethon cM position 186.7) on 6q27 (Figure 4C). Although there were fewer A3b2 families (Table 1), and hence less power to observe positive linkage using these families on their own, their removal from the analysis within each village clearly enhances the LOD scores obtained for the E3b1 pedigrees indicating that lack of linkage in these families was reducing the LOD scores. To evaluate the significance of our results given the possibility of type I errors due to over-relatedness of parents in the pedigrees [22,23], simulations were performed after adding inbreeding loops to the pedigrees based on the relationships between parents determined by the PREST analysis (Table 2). For the peak at D1S2868 in Um-Salala, a LOD score of 5.65 was never observed in 100,000 simulations, providing a point-wise empirical *p* value <1 × 10^−5^ that retains genome-wide significance [14]. This is consistent with the LOD score 4.3 (*p* = 4.3 × 10^−6^) obtained when the real data were re-analysed with inbreeding loops added to the pedigrees. For the peak at 6q27 in El Rugab, the point-wise empirical *p* value was <1 × 10^−4^ (10,000 simulations performed), again broadly in line with the LOD score 2.84 (*p* = 1.5 × 10^−4^) obtained for real data re-analysed with inbreeding loops added to the pedigrees. Given the increased locus-specific λ_S_ values observed with the stratified analyses, the susceptibility loci at 1p22 in Um Salala and 6q27 in El Rugab make a substantial contribution to the genetic susceptibility to disease in E3b1-tagged lineages in these villages. In our analysis of population substructure we also sequenced the hypervariable HVS-I region [24] of the mitochondrial genome for all families (Text S1). A diverse range of mitochondrial haplogroups was observed (Table S1) that showed little overlap across Y chromosome lineages within villages.

### No Evidence for Parent-of-Origin Effects

One interpretation of the Y chromosome stratified data could be parent-of-origin effects. We looked at this in two ways: (i) by comparing identity-by-descent allele-sharing for maternally versus paternally derived alleles across all affected relative-pairs in the pedigrees (Table 3); and (ii) by comparing GENEHUNTER-MODSCORE results under trait models with or without imprinting (Table 4). Neither method provided evidence for parent-of-origin effects at either 1p22 or 6q27. This means that susceptibility alleles introduced into villages by the small number of founding males and their families are being transmitted by both male and female parents. The multipoint GENEHUNTER-MODSCORE analysis (Table 4), which is a parametric analysis that maximizes the LOD score with respect to penetrances and disease allele frequencies, also demonstrates recessive inheritance for susceptibility alleles at both 1p22 and 6q27, consistent with transmission of disease alleles through both male and female parents.

**Table 3:**
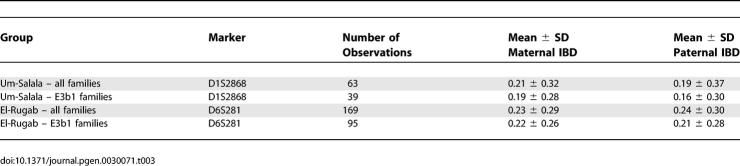
IBD Allele Sharing of Maternally Compared to Paternally Derived Alleles for Markers at the Peaks of Linkage on 1p22 and 6q27

**Table 4:**
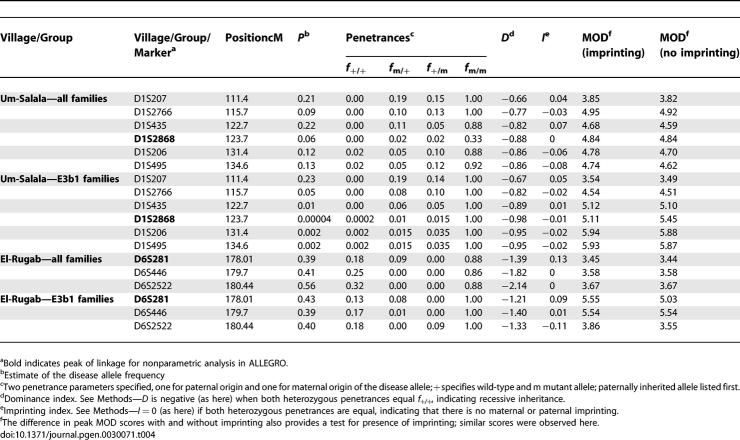
MOD Scores and Estimated Trait Model Parameters for Microsatellites on Chromosomes 1p22 for all Families and E3b1 Families from Um-Salala Village and 6q27 for all Families and E3b1 Families from El-Rugab Village

## Discussion

Here, we identified major lineage-specific genes at 1p22 and 6q27 controlling VL in adjacent Masalit villages in eastern Sudan. We hypothesized that chance events in the founding males and their families carried specific susceptibility alleles into each village, and that consanguineal marriage within patriarchal communities has enriched these alleles, leading to strong lineage-specific effects. This explains failure to replicate the major linkage peak on Chromosome 22q12 [13], although differences in disease phenotype and parasite strain could also contribute. In this setting in Sudan, Y chromosome haplotypes have provided tags for recently immigrant extended pedigrees that contribute to different linkages within, but not between, each village. This is consistent with the likelihood that Y chromosome haplotypes will only serve to mark the autosomes over a limited number of generations, in this case marking the autosomes carrying susceptibility alleles introduced by founders of extended pedigrees within each village. In this patriarchal society, mitochondrial haplotypes were more heterogeneous than Y chromosome haplotypes, and did not serve to mark the origin of the autosomes carrying the susceptibility alleles. Nevertheless, as expected for autosomal genes, susceptibility alleles introduced through the founding families tagged by Y chromosome haplotypes were inherited through both parents within the extended pedigrees. Our results demonstrate that an understanding of population substructure can contribute to the identification of disease susceptibility alleles in these recently immigrant African populations. In other settings, mitochondrial haplotypes, or a combination of Y and mitochondrial haplotypes, might both provide important markers of population substructure influencing the frequency and distribution of disease susceptibility alleles in different populations, as may a selection of autosomal markers.

Another important component of our study was that, in our analyses, we also took into account consanguinity and over-relatedness of parents within the pedigrees, which can lead to type I errors and inflated LOD scores [22,23]. It is possible that the LOD score of 3.5 (*p* = 3 × 10^−5^) reported by Bucheton et al. [13] for Chromosome 22q12 may also be inflated by consanguinity within the pedigrees used. To account for consanguinity, we employed a strategy similar to that used by Riaz et al. [25], who specifically selected highly inbred families to increase power in demonstrating genome-wide significance for linkage of stuttering to Chromosome 12 in 44 Pakistani families. In the absence of definitive information on the relationship between parents, the primary genome scan and additional markers used for refined mapping provide data from a large number of microsatellite markers that can be used to specify the level of inbreeding using PREST [16]. In addition, given the strong patriarchal society in this region of Sudan, we used Y chromosome haplotypes to specify families belonging to extended pedigrees within each village. Simulations performed after the addition of inbreeding loops to the pedigrees allowed us to determine a point-wise empirical *p* value <1 × 10^−5^ associated with the LOD score of 5.65, providing genome-wide significance for a visceral leishmaniasis susceptibility locus on Chromosome 1p22. Similarly, an empirical *p* value <1 × 10^−4^ was associated with the LOD score 3.74 for a locus at 6q27. Hence, we are confident that these two regions of the genome carry susceptibility genes for visceral leishmaniasis in this region of Sudan, and we have supporting evidence that a gene at 6q27 may also contribute to susceptibility to visceral leishmaniasis in Brazil [26].

Interesting candidate genes are located at 1p22, including *DR1,* which encodes the downregulator of transcription 1/TBP-binding negative cofactor 2, and inhibits transcription by binding to the TATA box binding protein [27] (TBP) located at 6q27. CIITA, the transactivator of major histocompatibility complex class II molecules in antigen presenting cells, requires the participation of, and is extremely sensitive to mutations in, TBP [28]. Other 1p22 candidates include glomulin (*GLMN,* also called FKBP-associated protein FAP48), which is antiproliferative for T cells[29], and growth factor-independent 1 *(GFI1),* which influences myeloid differentiation [30]. The Notch ligand delta-like 1 *(Drosophila) (DLL1)* and proteasome subunit beta-type 1 *(PSMB1)* are also at 6q27. Proteasome function is important in degradation of proteins by antigen-processing cells, which use the Notch pathway to instruct T cell differentiation [31]. Specifically, DLL1 induces T helper 1 cells to release interferon-γ, which is crucial for immune control of L. donovani [32]. Identification of the etiological genetic variants at 1p22 and 6q27 will contribute to our understanding of the complex interaction of genes and mechanisms associated with susceptibility to this important protozoan disease in humans.

## Materials and Methods

### Study sites.

The study was carried out in two villages, El-Rugab and Um-Salala, on the eastern bank of the River Rahad (Galabat Province, Gadaref State) in the heart of the endemic area in eastern Sudan (Figure 1). They are occupied by members of the Nilosaharan-speaking Masalit ethnic group who migrated to the area from El-Geneina in Darfur Province, western Sudan, principally following the great drought of the early 1980s, although a small number of founders (two brothers carrying the E3b1 haplotype and one sister and their respective families) first established Um-Salala village in 1969 [33]. Um-Salala village lies 40 km to the north of El-Rugab. At the December 1997 census the population of Um-Salala was 1,225 (566 males; 659 females) and that of El-Rugab was 2,084 (1039 males; 1045 females). These villages have been under annual surveillance for VL by the Institute of Endemic Diseases since the mid-1980s. In 1996 a treatment centre was established in the area by Médecins Sans Frontières. Epidemiological and further demographic details relating to the study site are described in detail elsewhere [7,33,34]. The annual incidence rate of VL cases is ~38.5/1,000 persons/y. The reported [33] mean ± SD age at diagnosis for clinical VL is 7.5 ± 5.1 y; all except one patient were <17 y of age. Isoenzyme typing of parasites isolated from cases during the 1995/1996 season revealed the presence of three zymodemes in this area, corresponding to L. donovani s.s., L. infantum and L. archibaldi [33]. Parasite isolates were not made from all cases used in this study. For this study the noninvasive buccal swab technique was used, and DNA extracted from the buccal swab buffer or amplified directly from the buffer using multiple displacement amplification (MDA, Molecular Staging; http://www.qiagen.com). Ethical approval for the study was obtained from the Institutional Review Board of the University of Khartoum, Khartoum, Sudan. Informed consent for sample collection was obtained from adults, and from the parents of children <18 y old.

### Families.

Multicase families with VL were ascertained from epidemiological and medical records of the Institute of Endemic Diseases and Médecins Sans Frontières. Families were pursued where records indicated two or more cases in the family. Households generally comprised a senior male with his sons, together with their (multiple) wives and families. When available, both fathers and mothers of families with VL cases were interviewed to determine pedigree structure, with DNA collected from those parts of the pedigree that were informative for linkage. A total of 69 nuclear families from 57 pedigrees were used at different stages of the study (Table 1). Of these, 97 individuals from 13 pedigrees with 20 nuclear families from Um-Salala village, and 123 individuals from 25 pedigrees with 28 nuclear families from El-Rugab village, were successfully genotyped and integrity of their families verified using the computer program PedCheck [35] for 360 out of 400 microsatellite markers typed for the primary genome scan. Additional families were used to complement the data at later stages of the study, but these did not have sufficient power to independently replicate linkage. Table 1 shows the numbers of individuals in families available for analysis after checking for genetic integrity (PedCheck [35]) within all families. Nuclear families with one affected offspring were always part of a larger pedigree, and so contributed to the linkage analysis, which compared allele-sharing across all relative pairs within the pedigree (cf. below).

### Diagnosis.

All of the individuals classified as affected in these families were diagnosed with clinical VL that responded positively to specific anti-leishmanial treatment. In this respect our affection status may be less severe than that used by Bucheton et al. [13], who reported that 2% to 5% of VL subjects died in spite of treatment. For our study, diagnosis of clinical VL was made on the basis of clinical, parasitological, and serological criteria as described [7,11,12,33]. At initial presentation, symptoms suggestive of VL included fever, often prolonged and not cyclical (differential diagnosis for malaria), pale continence of skin due to anaemia, weight loss, hepatosplenomegaly, and generalized lymphadenopathy. Specific antibody was measured using the direct agglutination test (DAT). Parasitological confirmation of cases was made by examination of Giemsa-stained lymph node aspirates. All clinical examinations were carried out by experienced clinical staff from the Institute of Endemic Diseases Leishmaniasis Research Group (83% of cases) or Médecins Sans Frontières (17% of cases). Only cases that were DAT positive, parasitologically confirmed by aspirates, and responded to specific anti-leishmanial treatment were included in the study. Subclinical forms of disease [33] were not included. The 57 families (Table 1) included 84 affected males (mean ± SD age at diagnosis 8.56 ± 4.02; range 2–16) and 87 affected females (mean ± SD age at diagnosis 8.86 ± 4.77; range 2–20). The affected parents reported in Table 1 were all historical cases >25 y of age at the time of sample collection.

### Genotyping.

For the primary genome scan, families were genotyped for the 400 markers that make up the Applied Biosystems medium density 10 cM resolution human index map. Data from 360 (90%) of these markers that were successfully genotyped (i.e., mean ± SD 79 ± 9% of individuals typed for every marker) and verified using PedCheck [35] were used for analysis of the primary genome scan. Refined mapping was performed on these families by successful genotyping and PedCheck [35] of 46 additional microsatellite markers in regions positive for linkage at *p* < 0.05. To complement data from the primary genome scan, 32 markers in positive regions from the ABI medium density map, and the 46 additional markers, were successfully genotyped and PedChecked [35] in additional families (Table 1). Tests for Hardy-Weinberg Equilibrium (HWE) were performed within Stata using genetically independent family members from the two villages. All markers used for linkage analysis were in HWE (data not shown).

### Y chromosome SNP typing.

SNaPshot assays using the ABI PRISM SNaPshot Multiplex System (http://www.appliedbiosystems.com) were designed to detect SNPs M02, M13, M40, M42, M78, M89, M118, M144, M145, M148, M171, M181, M215, and M224 as defined by Underhill et al. [20] and the Y Chromosome Consortium [21]. Carriage of the ancestral allele at M42, variant alleles at M144 and M13, and ancestral alleles at M118 and M171, classified individuals as belonging to haplotype A3b2 in haplogroup A [21]. Carriage of variant alleles at M42, M145, M40, M215, M78, and M224 classified individuals as belonging to haplotype E3b1 in haplogroup E [21]. Males from four families from El-Rugab carried variant alleles at M42, but were wild type at M145 and M181 which excluded them from haplogroups A, B, D, or E. On the basis of previously reported [20] haplogroups from Sudan, and the fact that haplogroup C does not occur in Africa, we placed these males into haplogroup J but we did not determine their specific haplotype. Lineages carrying the Y chromosome haplotypes A3b2 and E3b1 appeared equally susceptible to VL, as evidenced by the presence of 55.6% affected individuals in A3b2 families and 52.8% in E3b1 families for which phenotypic data were available for all sibs in the nuclear family. Ability to identify genes that control susceptibility to VL in the A3b2 haplogroup A families was limited since there were only four and two nuclear A3b2 families included in the primary genome scan families from El Rugab and Um-Salala, respectively. For refined mapping, there were eight and six A3b2 families, respectively (Table 1).

### Data analysis.

Nonparametric multipoint linkage analyses were performed in ALLEGRO [36], with results reported as allele sharing LOD scores [37] and plotted as sign(dhat)•LOD [36]. The S_pairs_ scoring function with 0.5 weighting was used to take account of differences in pedigree size [36]. The S_pairs_ scoring function compares allele-sharing identical-by-descent across all relative pairs within the extended pedigrees, including the singleton families within larger pedigrees. Unaffected members of pedigrees were included to assist ALLEGRO to infer missing parents' genotypes. Of the multicase nuclear families (*N* = 9 Um-Salala; *N* = 8 El-Rugab) where there was one missing parent, all had a minimum of two offspring and most (*N* = 6 Um-Salala; *N* = 5 El-Rugab) had three to six offspring. Two families with three (Um-Salala) and four (El-Rugab) offspring had both parents missing. Allele frequencies for the microsatellite markers were calculated separately for each stratified analysis in SPLINK [38], which uses unrelated individuals in the pedigrees to calculate frequencies. Information content for markers was estimated in ALLEGRO. All LOD scores reported are multipoint. Simulations (100) performed within ALLEGRO using data for a typical set of six linked polymorphic microsatellite markers (7–10 alleles; heterozygosity 0.73) showed that the primary genome scan family set (Table 1) across both villages had 100% power to detect a major gene at an allele-sharing LOD score = 3.00; *p* = 1.02 × 10^−4^, and >98% power to detect an allele-sharing LOD score = 3.95; *p* = 1.01 × 10^−5^. The separate El-Rugab and Um-Salala primary genome scan family sets had >94% and >79% power to detect a major gene at an allele-sharing LOD score = 2.07; *p* = 0.001, respectively. The primary scan plus additional families (refined map) had >96% and >97% power to detect a major gene at an allele-sharing LOD score = 2.07; *p* = 0.001 for El-Rugab and Um-Salala, respectively.

Parametric linkage analysis was performed using GENEHUNTER-MODSCORE [39], which maximizes the parametric LOD score with respect to penetrances and disease allele frequency, and is a further development of GENEHUNTER-IMPRINTING [40] based on the original GENEHUNTER version 2.1 [41]. Multipoint analysis was performed under trait models with and without imprinting. To allow for imprinting, two penetrance parameters (instead of one *f*_Het_ in the analysis for no imprinting) were specified for individuals who are heterozygous, one for paternal origin and one for maternal origin of the disease allele. The four penetrance parameters are *f*_+/+_, *f*_m/+_, *f*_+/m_, and *f*_m/m_, where + specifies the wild-type allele and m the mutant allele, and the paternally inherited allele is listed first. In conjunction with the MOD score, the analysis yields the penetrances and disease allele frequency *(p)* of the best-fitting trait model. The estimate of the disease allele frequency obtained by MOD-score analysis has the largest variance of all trait-model parameters [42], and can be higher than the true value because specifying a higher frequency can compensate for a general model misspecification and hence lead to robustness in a multipoint analysis [43]. The MOD-score analysis transforms penetrances to provide a dominance index *D,* and an index of imprinting *I. D* is positive (dominant model) if both heterozygote penetrances equal *f*_m/m_, negative (recessive model) if both heterozygote penetrances equal *f*_+/+_, and zero (semi-dominant or additive) if the average of the two heterozygote penetrances is halfway between the two homozygote penetrances. *I* is positive (maternal imprinting) or negative (paternal imprinting) if one heterozygote penetrance equals *f*_+/+_ and the other *f*_m/m_, and zero (no imprinting) if both heterozygotes penetrances are equal. A comparison of the difference in peak MOD scores obtained with or without imprinting provides an additional test for presence of imprinting [42]. Simulations performed by Weeks et al. [44] and Hodge et al. [45] show that a critical value of 3, used for LOD scores, should be adjusted by some value in the range of 0.3 to 1.0 to maintain a similar type I error. To account for the additional parameter of imprinting, a further adjustment of the critical value is necessary. Strauch et al. [42] propose that MOD scores >3.5 obtained with the imprinting parameter provide at least suggestive [14] evidence for linkage. Data for MOD scores are provided only where this critical value is achieved or exceeded.

A second evaluation of parent-of-origin effects or imprinting was determined by comparing sharing of maternally derived alleles identical-by-descent with paternally derived alleles identical-by-descent in affected relative pairs using the program MERLIN [46] that allows rapid analysis of allele sharing across extended pedigrees.

To estimate the contribution that specific loci make to the genetic component of disease susceptibility, we estimated [17] the locus-specific λ_S_ from the ratio of the expected (0.25) proportion of affected sibpairs sharing zero alleles identical-by-descent under no linkage to the observed proportion across all pedigrees, as determined using affected sibpair linkage analysis in GENEHUNTER [41].

The program PREST [16] was used to estimate degree of relatedness between individuals in our families by estimating the probabilities *P*_0_, *P*_1_, and *P*_2_ of two individuals sharing 0, 1, and 2 alleles identical-by-descent over the 360 microsatellite markers successfully genotyped in the primary genome scan, or 78 markers successfully genotyped in all families during refined mapping. Unrelated individuals should have probabilities 1, 0, and 0, respectively. Full sibs have *P*_0_ sharing probabilities of 0.25; half sibs plus first cousin 0.375; grandparent-child, avuncular or half-sib 0.50; double first-cousins 0.5625; first cousins or half-avuncular 0.75; half first cousins 0.875; and second cousins 0.9375. We therefore considered any relationship between parents in the families with *P*_0_ sharing probabilities <0.95 to be indicative of a consanguineal marriage, with predicted relationships as outlined in Table 2. On the basis of these predicted relationships, we used a procedure similar to that adopted by Riaz et al. [25], adding appropriate inbreeding loops to the pedigrees and carrying out 100,000 simulations for E3b1 pedigrees from Um-Salala using the refined Chromosome 1 map, and 10,000 simulations for E3b1 pedigrees from El-Rugab using the refined Chromosome 6 map, to determine empirical *p* values associated with the observed LOD scores for linkage to VL susceptibility.

### Web resources.

World Health Organization data on global distribution, prevalence, and incidence of visceral leishmaniasis can be found at http://www.who.int/tdr/diseases/leish/default.htm. Pregap and Gap4 programs are available at http://www-gap.mcs.st-and.ac.uk/Download/index.html. Information about the Applied Biosystems medium density map and microsatellite markers is available at https://products.appliedbiosystems.com/ab/en/US/adirect/ab?cmd=catNavigate2&catID=600770. Details of the SPLINK program are available at http://www-gene.cimr.cam.ac.uk/clayton/software/splink.txt.

## Supporting Information

Table S1 Summary of Mitochondrial Haplogroups in E3b1 and A3b2 Y Chromosome Lineages in Um-Salala and El-Rugab Villages in Eastern Sudan (63 KB DOC)

Text S1 Analysis of Mitochondrial Haplogroups (30 KB DOC)

### Accession Numbers

The Swiss-Prot database (http://expasy.org/sprot) accession numbers for the proteins discussed in this paper are DLL1, O00548; DR1, Q01658; GFI1, Q99684; GLMN, Q92990; PSMB1, P20618; and TBP, P20226.

## Abbreviations

PREST: Pedigree Relationship Statistical Test
VL: visceral leishmaniasis
