# Supplementary material for: Y Chromosome Lineage- and Village-Specific Genes on Chromosomes 1p22 and 6q27 Control Visceral Leishmaniasis in Sudan
Source: PLoS Genet. 2007 May 11;3(5):e71. doi: 10.1371/journal.pgen.0030071 (PMC1866354; doi:10.1371/journal.pgen.0030071)
Supplement: Supplementary file 1 [file pgen.0030071.sd001.doc]

**Text S1**

Supporting Material

**Analysis of mitochondrial haplogroups**

In our analysis of population substructure we also sequenced the hypervariable HVS-I region [1] of the mitochondrial genome for all family members. HVS-I was amplified by PCR using primers L15996 and H16401 [1] and sequenced using the ABI PRISM Big Dye Terminator Cycle Sequencing Ready reaction kit. Sequencing reaction products were run on an ABI3100 Sequence Analyzer (Applied Biosystems), and raw data assembled using the Pregap and Gap4 programs (Medical Research Council Laboratory for Molecular Biology, Cambridge, UK). In all cases, all mitochondrial sequences for offspring within nuclear families matched the maternal sequence. In Table S1, all SNPs indicated should be prefixed by 16 to indicate their real positions in the mitochondrial genome. Major subgroups shown in bold could sometimes be further subdivided by additional SNPs at positions indicated. The numbers in bold are the sums of the subgroups shown below. A letter after a SNP positions indicates a transversion mutation. Variant alleles at diagnostic SNP positions (Table S1) permitted assignment of mitochondrial sequences to 8 classifiable haplogroups (L1a1, L1b, L2a, L2c/d, L3b, L3d, L3e, L3f1) as defined by Salas et al. [2]. Stratification of the linkage analyses for each village by mitochondrial haplogroup did not provide any evidence for haplotype-specific effects. This was partly because the larger number of haplogroups meant that only one major haplogroup (L2a for El-Rugab, 16 nuclear families) had sufficient numbers of families to support a stratified analysis. In this case, 14 of the families belonged to Y halotype E3b1, which contributed to the linkage at 6q27. Within this group of families, there was additional evidence for sub-types of mitochondrial haplotypes (see Table S1), indicating that the Y halotype E3b1 was the true marker for the extended pedigrees contributing to this linkage rather than the L2a mitochondrial haplogroup. Additional SNPs in our population permit further subdivision of most of the mitochondrial haplogroups. Unless these represent recent mutation events (in mothers and then passed on to all children in the families), the major haplogroups are unlikely to represent true maternal relatedness within or between the pedigrees. In addition, 13 families had mitochondrial haplotypes that were not classifiable by HVS-I mutations alone, but each had unique SNPs in the HVS-I region. Overall the results demonstrate that identical haplotypes are rarely shared across pedigrees belonging to different Y-chromosome haplotypes (E3b1 versus A3b2) within villages, consistent with a lack of intermarriage between the major Y chromosome lineage. Heterogeneity in mitochondrial haplotypes meant that they were not useful as tags for lineages for the small numbers of families that we had in this study in Sudan.

**References**

1.	Vigilant L, Pennington R, Harpending H, Kocher TD, Wilson AC. Mitochondrial DNA sequences in single hairs from a southern African population. Proc Natl Acad Sci U S A 1989; 86:9350-9354.

2.	Salas A, Richards M, De la Fe T, et al. The making of the African mtDNA landscape. Am J Hum Genet 2002; 71:1082-111.
