# Supplementary material for: Y Chromosome Lineage- and Village-Specific Genes on Chromosomes 1p22 and 6q27 Control Visceral Leishmaniasis in Sudan
Source: PLoS Genet. 2007 May 11;3(5):e71. doi: 10.1371/journal.pgen.0030071 (PMC1866354; doi:10.1371/journal.pgen.0030071)
Supplement: Supplementary file 2 [file pgen.0030071.st001.doc]

Supplementary Table 1: Summary of mitochondrial haplogroups in E3b1 and A3b2 Y chromosome lineages in Um-Salala and El-Rugab villages in eastern Sudan.

|  | Number of pedigrees | | | |
| --- | --- | --- | --- | --- |
| Mitochondrial Haplogroup | Um-Salala | | El-Rugab | |
|  | E3b1 | A3b2 | E3b1 | A3b2 |
| L1a1: ref. SNPs 129,148,168,172,187,188G,189,223,239,311,320 | 4 | 2 | 0 | 1 |
| L1a1 | 1 | 0 | 0 | 1 |
| L1a1-261,362 | 1 | 0 | 0 | 0 |
| L1a1-93,209,278,293 | 1 | 0 | 0 | 0 |
| L1a1a | 1 | 0 | 0 | 0 |
| L1a1-294 | 0 | 1 | 0 | 0 |
| L1a1-344,357,390 | 0 | 1 | 0 | 0 |
| L1b: ref. SNPs 126,187,189,223,264,270,278,311 | 0 | 1 | 0 | 0 |
| L2a: ref. SNPs 223,278,294,(390) | 5 | 0 | 14 | 2 |
| α1* | 1 | 0 | 4 | 2 |
| α2-189 | 0 | 0 | 4 | 0 |
| β1-309 | 0 | 0 | 4 | 0 |
| β2-189,309 | 4 | 0 | 2 | 0 |
| L2c/d: ref. SNPs 223,278,(390) | 4 | 0 | 1 | 0 |
| L3b: ref. SNPs 124,223,278,362 | 1 | 0 | 1 | 0 |
| L3d: ref. SNPs 124,223 | 1 | 1 | 1 | 0 |
| L3d-111, 235,309,311 | 0 | 0 | 1 | 0 |
| L3d-148,257 | 1 | 0 | 0 | 0 |
| L3d-319 | 0 | 1 | 0 | 0 |
| L3e: ref. SNPs 223, 320 | 0 | 1 | 0 | 1 |
| L3e4: ref SNPs 223,264 | 0 | 1 | 0 | 0 |
| L3f1: ref. SNPs 209, 223, 292, 311 | 1 | 0 | 2 | 2 |
| L3f1 | 1 | 0 | 1 | 0 |
| L3f1-261G | 1 | 0 | 0 | 0 |
| L3f1-126 | 0 | 0 | 1 | 0 |
| L3f1-172A | 0 | 0 | 0 | 1 |
| L3f1-183C, 189, 193ins, 256 | 0 | 0 | 0 | 1 |
| Unclassified: | 5 | 3 | 2 | 3 |
| 223 only* | 2 | 1 | 0 | 2 |
| 223-209,304 | 1 | 0 | 0 | 0 |
| 223-209,309 | 1 | 0 | 0 | 0 |
| 223-286,320 | 0 | 1 | 0 | 0 |
| 223-209,234 | 0 | 1 | 0 | 0 |
| 223-179,256A,284,311 | 0 | 0 | 0 | 1 |
| 278,294,309,390 | 0 | 0 | 1 | 0 |
| 93,185,193ins,198,260,298,311 | 0 | 0 | 1 | 0 |
| 293C,311,355.362 | 1 | 0 | 0 | 0 |

* These haplogroups may be further subdivided by SNP variants outside the hypervariable regons.
